# Study of the Potential Endocrine-Disrupting Effects of Phenylurea Compounds on Neurohypophysis Cells *In Vitro*

**DOI:** 10.1155/2019/1546131

**Published:** 2019-02-10

**Authors:** Krisztián Sepp, Zsolt Molnár, Anna M. László, Tünde Alapi, László Tóth, Andrea Serester, Zsuzsanna Valkusz, Márta Gálfi, Marianna Radács

**Affiliations:** ^1^First Department of Internal Medicine, Faculty of Medicine, University of Szeged, Szeged, Hungary; ^2^Institute of Applied Science, Department of Environmental Biology and Education, Gyula Juhász Faculty of Education, University of Szeged, Szeged, Hungary; ^3^Department of Biometrics and Agricultural Informatics, Faculty of Horticultural Science, Szent István University, Budapest, Hungary; ^4^Department of Inorganic and Analytical Chemistry, Faculty of Science and Informatics, University of Szeged, Szeged, Hungary

## Abstract

Homeostatic disruptor agents, and endocrine disruptor compounds (EDC) specifically, can originate from agricultural and industrial chemicals. If they modify the adaptation of living organisms as direct (e.g., by altering hormone regulation, membrane functions) and/or indirect (e.g., cell transformation mechanisms) factors, they are classified as EDC. We aimed to examine the potential endocrine-disrupting effects of phenylurea herbicides (phenuron, monuron, and diuron) on the oxytocin (OT) and arginine-vasopressin (AVP) release of neurohypophysis cell cultures (NH). In our experiments, monoamine-activated receptor functions of neurohypophyseal cells were used as a model. *In vitro* NH were prepared by enzymatic (trypsin, collagenase) and mechanical dissociation. In the experimental protocol, the basal levels of OT and AVP were determined as controls. Later, monoamine (epinephrine, norepinephrine, serotonin, histamine, and dopamine) activation (10^−6^ M, 30 min) and the effects of phenylurea (10^−6^ M, 60 min) alone and in combination (monoamines 10^−6^ M, 30 min + phenylureas 10^−6^ M, 60 min) with monoamine were studied. OT and AVP hormone contents in the supernatant media were measured by radioimmunoassay. The monoamine-activated receptor functions of neurohypophyseal cells were modified by the applied doses of phenuron, monuron, and diuron. It is concluded that the applied phenylurea herbicides are endocrine disruptor agents, at least *in vitro* for neurohypophysis function.

## 1. Introduction

Nowadays, society is the most complex evolutionary scene, in which transactions are realized between human beings and their environment (including the atmosphere, hydrosphere, and lithosphere). In this relation, people modify their environment so as to maintain their existence (e.g., chemisation) [1]. Chemisation came to be as a result of the biological and ecological problems caused by the vast and ever increasing amounts of chemicals used in agriculture (pesticides, herbicides, and fertilizers) [2]. These chemicals are xenobiotics [3]. These days, persistent organic pollutants (POPs) are placed in the focus. Endocrine disruptor compounds (EDC) are a group of heterogeneous POPs, which can interfere with endocrine communication [4, 5]. Phenylurea herbicides as POPs inhibit photosynthesis and can be found as contaminants in surface water and groundwater [6, 7]. Phenylureas have been used in agriculture extensively because of their herbicidal properties [8]. Several of the chlorinated benzenes are known to be porphyrogenic, carcinogenic, and mutagenic in animals and humans [9]; EDC lead to anxiety and aggression in rats [10] and to predator avoidance and reproductive and social behaviors in fish [7].

Biological objects, in order to maintain their inner homeostatic balance, take inputs from their environment, which can induce system accommodation processes. This connection among the biotic and abiotic systems can materialize, e.g., via neuroendocrine communication [11]. The synthesis and release of endogenous oxytocin (OT) and arginine-vasopressin (AVP) can be induced by external stimuli from paraventricular, supraoptic, and suprachiasmatic nuclei. The nerve terminals in the posterior lobe of the pituitary gland store these peptide hormones and release them into blood vessels to provoke their biological functions [12, 13].

OT secretion induced by stimuli is inversely proportional to the inhibition of food intake and gastric motility and stimulates the release of somatostatin and gastrin [14]. On the other hand, the actions of OT modulate neuroendocrine reflexes to establish complex social behaviors [15]. Beyond its peripheral effects on reproductive organs, OT might have an effect on neurons responsible for the cognitive feelings of organisms [16]. It is known that AVP and OT affect social cognition, particularly the acquisition and retention of social recognition in rats and mice [17]. Both hypotension and extracellular fluid volume expansion appear to stimulate hypothalamic oxytocinergic neurons and OT secretion; in rats, OT increases blood pressure and pulse rate [18]. There are several published studies investigating the prosocial effects of OT in humans: behaviors that facilitate interpersonal relations, including trusting behavior, generosity, and cooperation [19, 20]. In the female reproductive system, the pregnant uterus is one of the traditional targets of OT [21]. Systematic OT could also act peripherally, stimulating smooth muscle cells of the male reproductive tract [20].

The peptidergic hormone AVP can also be released by external stimuli and plays roles as both neurotransmitter and neuromodulator in the brain [22]. AVP participates in the regulation of the cardiovascular system and water-electrolyte balance [23]. As an antidiuretic hormone, AVP regulates body osmolality, blood volume, and blood pressure [24]. From these studies, it is postulated that OT and AVP are crucial in the adaptation processes of living organisms [25].

In homeostatic balance, biogenic amines play an essential role in the regulation of AVP and OT. Biogenic amines (with the ability to activate or inhibit OT and AVP) are important in the response mechanisms to external inputs. It is well documented that neuronal systems contain biogenic amines, such as dopamine (DA), epinephrine (E), norepinephrine (NE), histamine (HA), and serotonin (5-HT). These mediators may contribute reinforcement information to influence memory performance [26, 27].

The monoamine-regulated OT and AVP release serves as a model for the study of neuroendocrine cell function [28–32].

The integrity of neuroendocrine regulation can be controlled by following endocrine function attractors in *in vitro* models [33, 34]. Such regulation attractor in the OT and AVP secretion *in vitro* model is the monoamine-activated (agonistic or antagonistic) hormone secretion result.

Homeostatic regulation is modified by environmental factors (e.g., presence of EDC). The effects of EDC factors do not impact on the baseline but on the regulation of attractors [35]. When studying subtoxic or low-dose *in vitro* models, the regulation attractor should be followed for a worthy effect.

Our purpose was to present contemporary research in connection with the complex environmental stressors and their effects on the equilibrium process of living systems. We investigated the potential endocrine-disrupting effects of phenylurea herbicides (phenuron-PU, monuron-MU, and diuron-DU) on the monoamine-regulated oxytocin and vasopressin release of neurohypophysis cell cultures.

## 2. Materials and Methods

### 2.1. Experimental Protocol

Male Wistar rats (Charles River, Isaszeg, Hungary, medically certified) from different litters (weighing 120-250 g, aged 4-6 weeks at the beginning of the research) were used for hypophysis cell culture model systems. The animal care and research protocols were in full accordance with the guidelines of the University of Szeged, Hungary. During the research period, rats were kept under controlled relative air humidity of 55-65% and 22 ± 2°C ambient temperature. Experimental animals lived under automated diurnal conditions (12 h darkness and 12 h light system) in groups of 10 animals. Standard pellet food and tap water were available *ad libitum*. After pentobarbital anaesthesia (4.5 mg/kg b.w. Nembutal, Abbott, USA), the animals were killed and decapitated. Neurohypophysis and adenohypophysis tissues were separated under a preparative microscope.

The neurohypophysis tissue was digested enzymatically with 0.2% trypsin (Sigma, Germany) in phosphate-buffered saline for 60 min and with 0.05% collagenase (Sigma, Germany) for an additional 60 min at 37°C. The enzymatic hydrolysis was stopped by the addition of 100 *μ*g/mL trypsin inhibitor (Sigma, Germany). Mechanical disintegration of the tissue was performed on nylon blutex sieves (pore sizes 100, 80, and 48 *μ*m in series). Cultures were controlled for both viability (>95%; trypan blue exclusion) and function, and the cell density was determined to be 2 × 10^5^ cells/mL. The dispersed cells were placed onto 24-well plastic plates (Costar, USA) coated with 5% rat-tail collagen (Sigma, Germany). The starting cell density was 2 × 10^5^ cells/mL of medium (Dulbecco's modified Eagle's medium; Sigma, Germany) supplemented with 20% foetal calf serum (Gibco, USA) and 100 *μ*g/L PENSTREP (Sigma, Germany). The cell cultures were maintained at 37°C in a humidified atmosphere of 5% CO_2_ in air. The culture medium was changed every 3 days. The 14-day-old neurohypophysis primary cell cultures were standardised by immunohistochemical methods (by relative incidence/unit plate-area of IR content OT or AVP cells), and the OT and AVP time-release kinetic activity was determined.

After these procedures, the basal OT and AVP levels were measured in neurohypophysis cell cultures.

### 2.2. Cell Culture Treatment Protocol (Figure 1)

The control samples were untreated and served as self-controls, which showed the basal release (60 min) of OT and AVP in NH.

**Figure 2:**
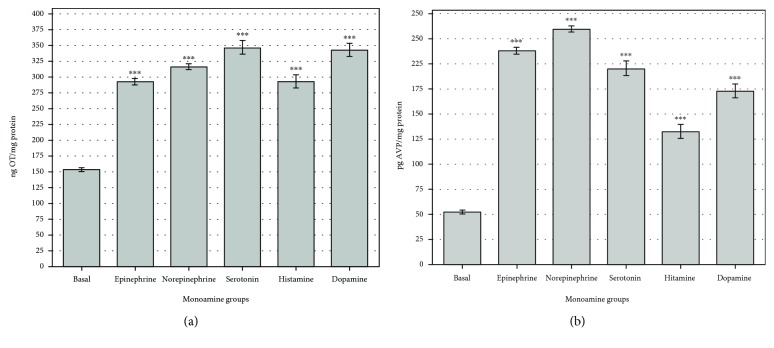
The effect of monoamines on OT (a) and AVP (b) release in neurohypophysis cell cultures. Pairwise comparisons verified the regulation cycle: all monoamine [10^−6^ M] groups increased significantly (^∗∗∗^*p* < 0.0001) compared to the basal regulation in control OT (*n* = 12) and AVP (*n* = 10). All data presented as mean ± Std.

The effects of PU, MU, and DU added to the NH for 60 minutes at concentration of 10^−6^ M were examined one by one [36]: PU CAS registry number: 101-42-8, DU CAS registry number: 330-54-1, and MU CAS registry number: 150-68-5 (Sigma, Germany). In our earlier studies, dose-dependent kinetics of phenylurea agents were determined, and the experimental doses were selected, because the saturation of receptor-binding sites depends on the affinity and number of receptor-binding molecules. Monoamine-activated G-protein receptors in NH were treated with 10^−6^ M (E, NE, 5-HT, HA, and DA) (Figures 2(a) and 2(b)) for 30 min [28–32] and the same concentration (10^−6^ M) of phenylurea agents.

The combined treatment groups of the NH were treated firstly with monoaminergic compounds (for 30 min), then phenylurea compounds (for 60 min): (1) E + PU, NE + PU, 5-HT + PU, HA + PU, and DA + PU; (2) E + MU, NE + MU, 5-HT + MU, HA + MU, and DA + MU; and (3) E + DU, NE + DU, 5-HT + DU, HA + DU, and DA + DU.

The OT and AVP contents were detected in the supernatant media. From the supernatant media, 500 *μ*L samples were removed by Gilson pipette at appropriate times and stored at −80°C until peptide radioimmunoassay (RIA) was performed to measure OT and AVP [10, 37].

A modified Lowry method [38] and Pierce BCA Protein Assay Kit (Thermo Fisher Scientific Inc., Rockford, USA) were used for the determination of total protein content.

### 2.3. Statistical Analysis

Pooled samples of neurohypophysis cell cultures (12 lots) were measured for OT and AVP hormone release in different EDC groups (control, PU, MU, and DU) by monoamine regulation (basal, E, NE, 5-HT, HA, and DA) in rats (*n* = 10 or 12 per group). Data were analyzed using mixed models [39–41]. The monoamine regulation cycle was verified in mixed models for the comparison of 6 monoamine levels in the control groups for OT and for AVP. In the random intercept model, monoamine was used as the fixed factor and the lots as the intercept.

For the two investigated hormone data (OT, AVP), mixed models were applied with EDC and monoamine as fixed effects and random intercept for the lots. In the analysis models, the reference group was the control (no EDC treatment) basal (no monoamine) group.

Restricted maximum likelihood estimation and the Kenward-Roger method for adjusting the degrees of freedom were applied in all models with unstructured covariance matrix. Pairwise comparisons were estimated by least squares means using Šidák *p* value adjustment. Model residuals were checked for normality assumptions.

Statistical analyses were performed in SAS (Version 9.3 SAS Institute Inc., Cary, NC, USA), where *p* values of <0.05 were considered to indicate statistical significance [42].

## 3. Results

Stability of homeostatic systems can be followed by examining the endocrine regulation cycle. Therefore, the monoamine-activated OT/AVP release cycles were modelled by NH *in vitro*.

**Figure 3:**
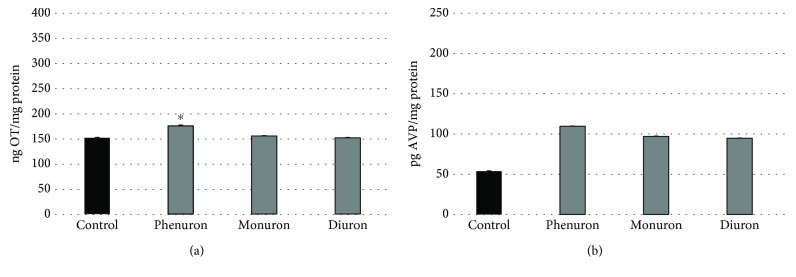
The effect of phenylureas [10^−6^ M] on OT (a) and AVP (b) secretion in neurohypophysis cell cultures. (a) *n* = 12, mean (ng OT/mg protein) ± Std; ^∗^*p* < 0.01. (b) *n* = 10, mean (pg AVP/AVP protein) ± Std.

The result of PU, MU, and DU alone on neurohypophysis cell culture can be seen in Figures 3(a) and 3(b). OT release was not modified significantly by treatment with MU or DU, but PU (176.67 ± 1.25 ng OT/mg protein) can modulate it compared to the control (153.13 ± 0.63 ng OT/mg protein). AVP release did not show significant difference in the case of phenylurea agents.

**Figure 4:**
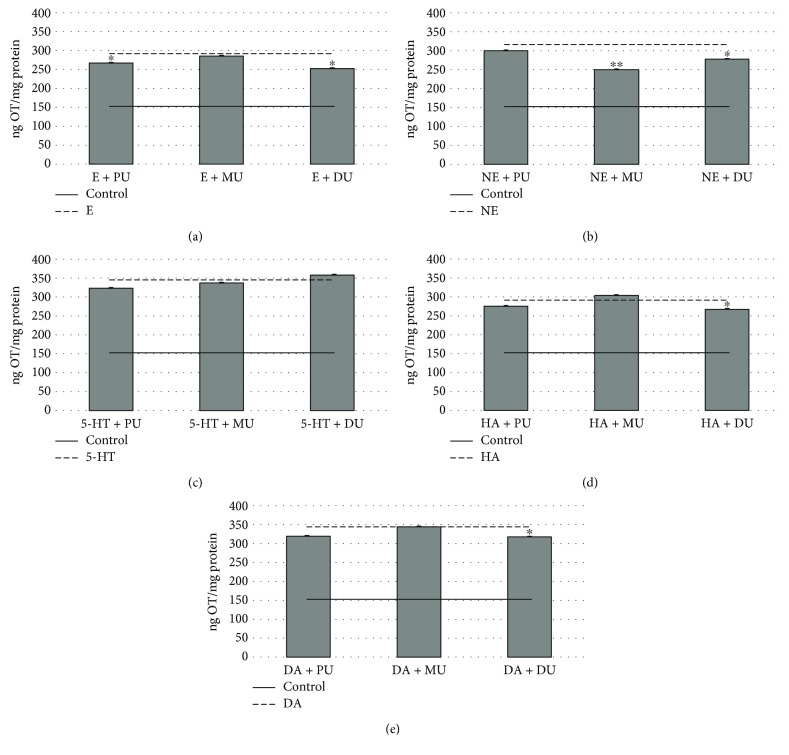
The effect of 10^−6^ M phenylureas on OT release in NH via 10^−6^ M monoamine-activated receptor functions. *n* = 12; mean (ng OT/mg protein) ± Std; ^∗^*p* < 0.01 and ^∗∗^*p* < 0.001. OT: oxytocin; PU: phenuron; MU: monuron; DU: diuron; E: epinephrine; NE: norepinephrine; 5-HT: serotonin; HA: histamine; DA: dopamine.

**Figure 5:**
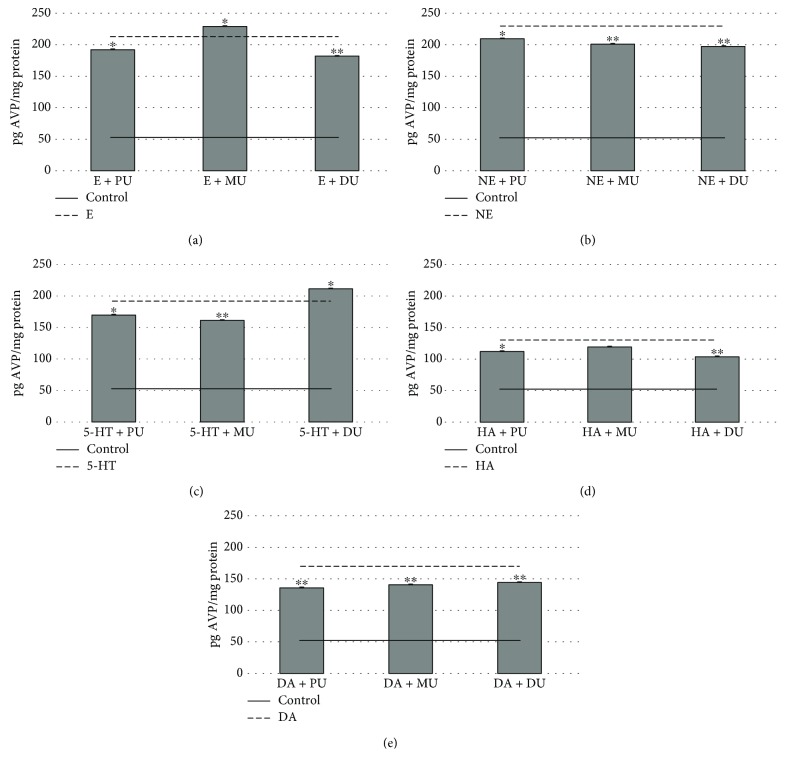
The effect of 10^−6^ M phenylureas on AVP release in NH via 10^−6^ M monoamine-activated receptor functions. *n* = 10; mean (pg AVP/mg protein) ± Std; ^∗^*p* < 0.01 and ^∗∗^*p* < 0.001; AVP: arginine-vasopressin; PU: phenuron; MU: monuron; DU: diuron; E: epinephrine; NE: norepinephrine; 5-HT: serotonin; HA: histamine; DA: dopamine.

OT and AVP release is an indicator of the monoamine-activated receptor cycle of neurohypophysis cells. The changing of these mechanisms was investigated using phenylurea agents (Figures 4 and 5). E (292.81 ± 1.49 ng OT/mg protein) activated OT secretion (153.13 ± 0.63 ng OT/mg protein) (Figure 4(a)) was significantly decreased by PU (268.71 ± 1.07 ng OT/mg protein) and DU (255 ± 0.10 ng OT/mg protein).  Figure 4(b) shows the effects of phenylureas on NE activation. MU (252.47 ± 4.75 ng OT/mg protein) and DU (277.09 ± 1.10 ng OT/mg protein) significantly reduced the effect of NE (316.55 ± 1.36 ng OT/mg protein). OT exocytosis induced by 5-HT is shown in Figure 4(c). The used phenylurea agents caused only discrete changing of OT release in NH. In the case of HA (292.91 ± 3.16 ng OT/mg protein), only the combination with DU (264.89 ± 1.39 ng OT/mg protein) reduced the OT release significantly as seen in Figure 4(d).  Figure 4(e) shows OT secretion for DA (342.06 ± 2.65 ng OT/mg protein), and again, only the combination with DU (318.72 ± 1.74 ng OT/mg protein) decreased it significantly.

In the case of AVP hormone, the E (212.41 ± 0.59 pg AVP/mg protein) activation was modified significantly by PU (193.01 ± 0.54 pg AVP/mg protein), DU (182.94 ± 0.59 pg AVP/mg protein), and MU (230.18 ± 0.55 pg AVP/mg protein) (Figure 5(a)). NE (231.33 ± 2.41 pg AVP/mg protein) induced AVP release (Figure 5(b)) was reduced by PU (211.96 ± 0.49 pg AVP/mg protein), MU (203.95 ± 0.64 pg AVP/mg protein), and DU (201.19 ± 0.52 pg AVP/mg protein). The effects of 5-HT (194.11 ± 0.49 pg AVP/mg protein) on NH cell AVP release (Figure 5(c)) were decreased by PU (172.88 ± 0.43 pg AVP/mg protein) and MU (163.70 ± 0.54 pg AVP/mg protein) and increased by DU (214.44 ± 0.90 pg AVP/mg protein). In Figure 5(d), it can be seen that the effect of HA (131.65 ± 0.53 pg AVP/mg protein) induced AVP release was diminished by PU (113.55 ± 0.72 pg AVP/mg protein) and DU (105.48 ± 0.89 pg AVP/mg protein). The DA (172.11 ± 0.94 pg AVP/mg protein) receptor-mediated AVP release (Figure 5(e)) in NH was significantly depressed by PU (138.72 ± 0.65 pg AVP/mg protein), MU (143.19 ± 0.71 pg AVP/mg protein), and DU (146.34 ± 1.24 pg AVP/mg protein).

## 4. Discussion and Conclusions

Bretaud et al. pointed out that exposures to sublethal concentrations of carbofuran, DU, and nicosulfuron have remarkable effects on acetylcholinesterase activity in the brain of goldfish (*Carassius auratus*) [43]. It is described that the herbicide MU inhibits growth and heterocyst formation in the nitrogen-fixing cyanobacterium *Nostoc muscorum* [44]. The results of Federico et al. showed direct genotoxic activity caused by the effects of PU, DU, and difenoxuron, as is demonstrated by the increasing number of chromosomal aberrations and sister chromatid exchange in Chinese hamster ovary and epithelial cell lines [45]. However, there is a serious lack of information on the effects of these agents on the endocrine cells.

In neuroendocrine communication, monoamines are known to be relevant regulators for the hormone release. Earlier studies have established that *α*_1_ receptors are involved in the E-induced increase in OT and AVP secretion, while the *β*_2_ receptor has a role in NE-mediated OT and AVP secretion. 5-HT_1_ and 5-HT_2_ receptors are involved in the 5-HT-induced increase in OT secretion while only 5-HT_2_ receptor has a role in the AVP secretion in NH. Our results show that H_1_ and H_2_ receptors are involved in the HA-induced increase in OT and AVP release in neurohypophysis cell cultures. D1 receptors are involved in the DA-induced enhancement of OT and AVP secretion. All mentioned monoamine receptors are G-protein-coupled, and cAMP-mediated mechanisms are induced in cells. OT and AVP hormone secretion is realized through protein kinase-C mechanisms (Figures 2(a) and 2(b)) [20, 46, 47].

In our *in vitro* model system, the EDC effects were detected by following hormone release via the disturbance of monoamine receptor function. The phenylureas alone did not show effects on basal hormone secretion of NH (Figure 3(b)), expect for PU on OT release (Figure 3(a)). However, the neurohypophysis cell function was modified by the combined treatment of monoamines and PU, MU, and DU. With regard to OT secretion, PU has an effect in epinephrine activation (Figure 4). MU has a stronger effect in NE activation. E, NE, HA, and DA activation was decreased significantly by the effect of diuron. In the phenylurea homologue line, the used DU was twice chloro substituted which can explain the difference in the endocrine effect [48, 49].

With regard to AVP release, the effects of used phenylureas were more expressed in all monoamine activation (Figure 5). PU had a significant effect on E, NE, 5-HT, HA, and DA monoamine activation. E activation was enhanced, and NE, 5-HT, and DA activation was reduced significantly by the used dose of MU (with the exception of HA activation). DU increased serotonin activation and decreased E, NE, HA, and DA monoamine activation significantly. These results are interesting in the case of AVP (as neurotransmitter and neuromodulator) because this hormone has an essential role in the endocrine-regulating body of osmolality, blood volume, blood pressure, cell contraction, and proliferation [23, 50]. The abovementioned hormones play a crucial role in horizontal homeostatic regulation; thus, altering the secretion of OT and AVP may influence the adaptation processes of living organisms to the external environment.

These findings confirm the classification of phenylureas as EDC. The environmental pollution by EDC may have chronic and/or acute effects on living organisms. Our results showed that the applied doses of phenylurea herbicides have direct and indirect effects on biological systems. Presumably, these phenylurea herbicides can disturb the association of monoamines to their receptors. The applied EDC per se play the role of possible modulator agents. In this context, neurohypophysis function is a crucial conditioning process.

E-, NE-, HA-, 5-HT-, and DA-activated hormone release mechanisms are mediated G-protein-coupled receptors [28, 29, 51]. These processes induce G_q_/G_11_ proteins, which, in turn, activate phospholipase-C, which catalyses the hydrolysis of phosphatidylinositol 4,5-bisphosphate to yield inositol triphosphate and diacylglycerol [52–54]. These signal transducers can enhance the intracellular [Ca^2+^] which is essential for OT and AVP expression. A detailed understanding of the mechanisms of hormone expression and functions is required to fully understand the workings of EDC, particularly the possibility that low doses of PU, MU, and DU might interfere with monoaminergic receptors.

In conclusion, the effects of phenylurea herbicides on monoamine-induced OT and AVP release show an EDC character based our results of an *in vitro* model system.

## Figures and Tables

**Figure 1:**
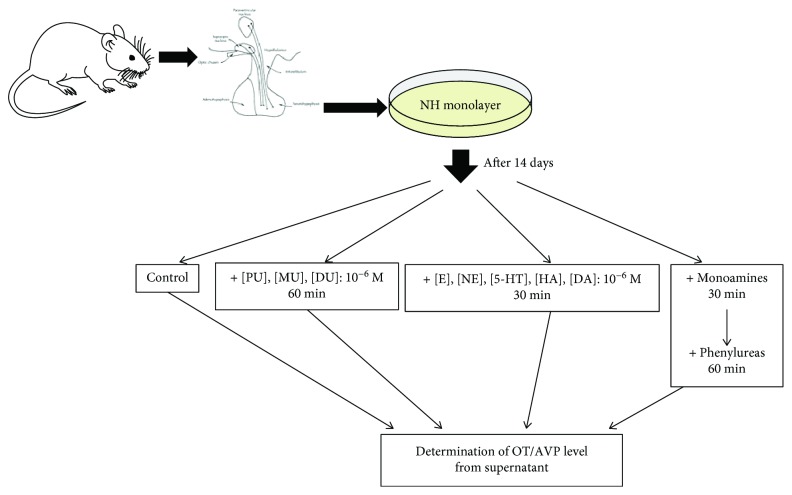
The *in vitro* treatment protocol. PU: phenuron; DU: diuron; MU: monuron; E: epinephrine; NE: norepinephrine; 5-HT: serotonin; HA: histamine; DA: dopamine; OT: oxytocin; AVP: arginine-vasopressin.

## Data Availability

Requests for data, after initial publication, will be considered by the corresponding author.
